# A Qualitative Study of Barriers to Accessing Water, Sanitation and Hygiene for Disabled People in Malawi

**DOI:** 10.1371/journal.pone.0155043

**Published:** 2016-05-12

**Authors:** Sian White, Hannah Kuper, Ambumulire Itimu-Phiri, Rochelle Holm, Adam Biran

**Affiliations:** 1 Department for Disease Control, London School of Hygiene and Tropical Medicine, Keppel Street, WC1E 7HT, London, United Kingdom; 2 International Centre for Evidence in Disability, London School of Hygiene and Tropical Medicine, Keppel Street, WC1E 7HT, London, United Kingdom; 3 Department of Education and Teaching Studies, Mzuzu University, Private Bag 201, Karonga Road, Mzuzu 2, Malawi; 4 Mzuzu University Centre of Excellence in Water and Sanitation, Mzuzu University, Private Bag 201, Karonga Road, Mzuzu 2, Malawi; University of Perugia, ITALY

## Abstract

Globally, millions of people lack access to improved water, sanitation and hygiene (WASH). Disabled people, disadvantaged both physically and socially, are likely to be among those facing the greatest inequities in WASH access. This study explores the WASH priorities of disabled people and uses the social model of disability and the World Health Organization’s International Classification of Functioning, Disability and Health (ICF) framework to look at the relationships between impairments, contextual factors and barriers to WASH access. 36 disabled people and 15 carers from urban and rural Malawi were purposively selected through key informants. The study employed a range of qualitative methods including interviews, emotion mapping, free-listing of priorities, ranking, photo voice, observation and WASH demonstrations. A thematic analysis was conducted using nVivo 10. WASH access affected all participants and comprised almost a third of the challenges of daily living identified by disabled people. Participants reported 50 barriers which related to water and sanitation access, personal and hand hygiene, social attitudes and participation in WASH programs. No two individuals reported facing the same set of barriers. This study found that being female, being from an urban area and having limited wealth and education were likely to increase the number and intensity of the barriers faced by an individual. The social model proved useful for classifying the majority of barriers. However, this model was weaker when applied to individuals who were more seriously disabled by their body function. This study found that body function limitations such as incontinence, pain and an inability to communicate WASH needs are in and of themselves significant barriers to adequate WASH access. Understanding these access barriers is important for the WASH sector at a time when there is a global push for equitable access.

## Introduction

The Sustainable Development Goals (SDGs) aim to provide access to improved sanitation and improved water sources *to all* by 2030 [1]. This will require services to be delivered to the hardest to reach, the poorest and those whose water, sanitation and hygiene (WASH) needs are currently not addressed by mainstream programming. Disabled people are reported to be at increased risk of having inadequate access to WASH facilities [2, 3]. The World Disability Report estimates that 15% of the world’s population are disabled [3]. Consequently, the new SDG will not be met unless access to WASH is improved for disabled people.

Both disability and WASH access are related to poverty. It is estimated that 80% of disabled people live in the developing world, [4] and in the poorest quintiles of low-income-country populations as many as 1 in 5 individuals are disabled [4]. Although the definition of ‘disabled’ used here is unclear, the higher prevalence of disability in low-income settings can be accounted for in three ways. Firstly there are inadequate health care and rehabilitative services in these countries. Secondly, the incidence of impairment may be higher due to unsafe environments and prevalent infectious diseases. Lastly, there are greater environmental and social barriers which result in individuals being more disabled by their impairments. Households in the poorest wealth quintile are also 5.5 times more likely to lack access to an improved water source and 3.3 times more likely to lack adequate sanitation compared with the highest wealth quintile in the same country [5–7].

The term ‘disability’ is defined and used in different ways reflecting differences in theoretical perspective. Throughout this paper the term ‘impairment’ is used to denote a loss or limitation in physiological or cognitive function (e.g. visual or hearing impairment, or impairment of mental functions). The terms ‘disabled person’ or ‘disability’ are used to denote a reduction in the ability of an individual to perform activities or participate fully in society (e.g. take part in employment). The discussion of disability and WASH has been informed by the social model of disability which views disabled people as an oppressed group who have “disability imposed on them” by society through exclusion and limitations on an individual’s opportunity to participate [8]. The social model arose in opposition to the medical model of disability which conceptualised disability as a consequence of an individual’s impairment and proposed medical interventions as the primary means to address this [9]. An alternative way of understanding disability has been put forward in the World Health Organization’s International Classification of Functioning, Disability and Health (ICF) framework [10]. The ICF framework aims to synthesise elements of the social and medical models, recognising disability as a complex phenomenon requiring interventions that operate at different levels, ranging from the medical to the socio-political [11]. While the social model sees disablement as a consequence of external factors in the environment, the ICF model views it as the consequence of an individual’s personal characteristics, such as body function, their social and demographic characteristics and characteristics of their social and physical environment, and as such is a bio-psycho-social model of disability.

Following the social model of disability the barriers disabled people face in accessing WASH have been categorised into three qualitatively distinct types [4, 12–17]. Physical barriers comprise environmental factors such as uneven terrain or muddy ground, as well as barriers associated with built infrastructure, such as steps or inappropriate pump handles. Institutional barriers include policies and institutions within the WASH sector that overlook the needs of disabled people or prevent their participation in the design and delivery of WASH programmes [12]. Lastly, social barriers arise through interaction with other people and result from cultural beliefs or practices. Social barriers may include beliefs that disability is due to a curse, and consequently that disabled people should be kept away from WASH facilities. They may also include overprotective parental attitudes preventing a disabled child from fully participating in community life. This taxonomy of barrier types suggests the challenges disabled people face when accessing WASH are largely due to factors in the external environment. The ICF framework, consistent with its incorporation of perspectives from the medical model, recognises the role of factors that are intrinsic to an individual with impairment.

### Purpose of study

Planning for and addressing the needs of disabled people within WASH programmes will be better achieved if based on a sound understanding of the needs and priorities of disabled people, the barriers they face and their coping strategies. The primary objective of this study was to understand barriers faced by disabled people in Malawi, across a range of WASH practices and to explore how these vary with differences in impairment, setting, gender and socio-economic status. The study also explored the extent to which WASH access is regarded as a problem by disabled people.

## Methods

### Study site

The study took place in 10 Traditional Authorities (TAs) and townships in Malawi (See Table 1). These were purposively selected from across the Northern, Central and Southern regions. Data collection was done through local languages and with the support of three field assistants from the Federation for Disabled People in Malawi (all of them disabled people). Data collection took place between July and October 2014.

**Table 1:** Study sites.

Traditional Authorities (TAs) and townships where the research took places	Region of Malawi	Type of region
Zolokere	Northern	Rural
Chikulamayembe	Northern	Rural /Peri-urban
Katumbi	Northern	Rural
Chisovya	Northern	Rural
Rumphi Boma	Northern	Peri-urban
Lilongwe Area 36	Central	Urban
Kuntaja	Southern	Rural
Machinjiri	Southern	Peri-urban
Bangwe	Southern	Urban
Ndirande South Ward	Southern	Urban

### Sample and recruitment

A sample of 36 disabled individuals and 15 caregivers (each caring for one of the 36 disabled individuals) was recruited. Respondents were purposively sampled to reflect a range of impairments as well as variety in location and socio-demographic characteristics. The sample was not intended to be statistically representative. Respondents in each TA were identified through a two-step process beginning with consultation of ‘key informants’ who included village chiefs, representatives of Disabled Persons Organisations (DPOs), local social welfare officers and other service providers. Following the Washington Group short list of screening questions [18], key informants were asked to identify individuals in their community who they thought would experience ‘some difficulty’, ‘a lot of difficulty’ or be ‘unable to do’ any of six specified activities: seeing, hearing, walking, remembering/understanding, communicating and self-care. Lists of up to 20 names, along with descriptions of each individual’s impairment type, were generated by key informants in each region. Based on these lists the research team purposively selected participants based on impairment type, gender, age and geographical location. The second step in the process involved the research team confirming the eligibility (determined by having at least ‘some difficulty’ in doing at least one of the six activities) of each of the purposively selected participants using the same short set of questions with the participant and/or the caregiver.

### Data collection

The study employed a range of participatory and qualitative methods. These are described in Table 2. These methods drew on tools developed by the Water, Engineering and Development Centre (WEDC) at Loughborough University[19], WaterAid, The Centre for Evidence in Disability at the London School of Hygiene and Tropical Medicine and the Leonard Cheshire Disability and Inclusive Development Centre (LCDIDC) at University College London. All research tools are available at: http://ehg.lshtm.ac.uk/wash-disability.

**Table 2:** Summary of methods.

Description	Purpose	Sample Characteristics	Sample Size
**Free Listing and Ranking:** Respondents listed the most difficult parts of their day or the things that they would most like to do independently. Respondents then ranked the difficult parts of the day according to their perceived severity of the problem represented.	Understand the relative importance of WASH challenges for respondents.	Disabled people and carers for people with intellectual impairments.	6 disabled people 3 caregivers
**PhotoVoice / PhotoVoice Ranking**: Respondents were taught how to use cameras. They were asked to take 5 photos representing the most difficult parts of their day or the things they would most like to do independently. In the PhotoVoice ranking exercise respondents ranked the difficult parts of the day according to the perceived severity of the problem represented.	Understand where WASH issues fit within the larger context of issues faced by disabled people and express these issues through their own perspective and creativity.	Respondents with requisite intellectual and motor abilities to handle a camera and fulfil the activity.	5 disabled people
**Video Observation:** Video of participant’s routine domestic activities carried out for three hours commencing early morning when respondents first awoke.	Understand WASH related activities within daily routines and particularly the issues faced by people for whom communication is difficult.	3 people with intellectual impairments who had a limited ability to answer questions. 3 respondents with a mobility impairment.	6 disabled people
**WASH Demonstrations:** Respondents re-enacted routine WASH related activities (always done fully clothed).	Rapid assessment of WASH access barriers to inform subsequent interview.	All	36 disabled people
**Emotion Mapping**: Respondents draw a picture of their house and the surrounding area including their toilet, bathing place and nearest water point. Two pictures, one a happy face and one a sad face, were used by the participant to indicate locations where they felt happy or sad.	To map emotions associated with WASH locations. Primarily ice-breaking prior to interviews.	Children and respondents with intellectual impairments.	3 disabled people
**In-depth Interviews:** Interviews were undertaken in the respondent’s home and lasted 15 minutes to 1 hour. In some cases interviews had to be done through a sign language interpreter or with the aid of a family member. Interviews were informed by WASH demonstrations allowing probing around specific practices observed.	Understand current WASH practices and the effects on lives and livelihoods.	All respondents with requisite intellectual abilities (3 individuals were not able to participate in this method)	33 disabled people 15 caregivers

### Data management and analysis

In-depth interviews were audio recorded, translated, transcribed and then thematically analysed by the lead author with the aid of nVivo 10, following a six-step analysis process [20]. Data were also captured via photographs and video. Footage from video observations and WASH demonstrations were annotated and coded with other data. Responses generated from the free listing and ranking of challenges were entered into a spreadsheet and coded together with interview transcriptions. The same was done for Photovoice rankings. Data were anonymised and categorised by respondent gender, age, geographical location, employment status and impairment. Coding was done through a deductive, ‘top down’ analysis [21] based on the study objectives. This included coding of responses by barrier types, priorities and type of WASH activity. Emergent themes were identified across the entire dataset and refined. Quotes were selected to illustrate themes.

### Ethics approval and consent

This study received ethical approval from the London School of Hygiene & Tropical Medicine and the Republic of Malawi National Committee of Research in the Social Sciences and Humanities. Participation was on the basis of informed, written consent (or use of a thumb mark in the case of illiteracy). Guardians provided consent for individuals under 18. Three participants with intellectual impairments were unable to give independent consent. In these cases consent was obtained from their primary caregiver. Wherever possible, interviews with caregivers or disabled participants were done separately and in private in the participant’s home.

## Results

### Characteristics of sample

The sample (described in Table 3) was 55% male. Participants ranged in age from 8 to 87 years (median age: 37.5). Participants were predominantly unemployed or in informal work (58%). Of 5 school-aged participants only 1 attended school. Of the 15 caregivers, 14 were women and all were family members, the majority being mothers or grandmothers of disabled children. Carers were interviewed if they played a role in aiding WASH access. In the majority of cases this was found to be with participants who had intellectual impairments and profound physical impairments.

**Table 3:** Characteristics of sample.

**Total Sample**	
Disabled participants	36
Age	
Range	8–87 years
Mean age	36 years
Under 18 years	5
Over 18 years	31
**Gender**	
Male	20
Female	16
**Geographical location**	
Urban	13
Peri-urban	11
Rural	12
**Employment status**	
Regular formal employment	9
Informal work	10
Unemployed	11
Student	1
School age but not at school	3
Retired	2
**Nature of impairments/health condition** (several individuals had more than one impairment)	
**Physical impairment**	**31**
Paralysis	7
Limb impairment	4
Limb loss	4
Epilepsy	4
Joint pain and arthritis	3
Albinism	2
Cerebral palsy	2
Spinal curvature	2
Restricted growth (dwarfism)	1
Peripheral neuropathy	1
Blood disorders (sickle cell anaemia)	1
**Visual impairment**	**6**
**Hearing impairment**	**5**
**Cognitive/intellectual impairments**	**8**
Intellectual impairment	5
Dementia	2
Mental health challenges	1

### Prioritisation of WASH challenges

A sub-sample of six respondents with physical or sensory impairments and three carers of individuals with intellectual impairments free-listed and ranked the biggest challenges they faced in daily living. A further five respondents with physical or sensory impairments identified and ranked their biggest challenges using Photovoice. A total of 65 challenges of daily living were generated by the disabled respondents and 16 challenges were generated by the three caregivers.

Two of the disabled respondents did not list any WASH related activities. Both had previously found bathing and toileting to be such a profound challenge that they had invested in adaptations to overcome these barriers. They explained:

“Where I used to live these things were very difficult for me. So now that we have our own house I made sure that there was a nice toilet like this and piped water. But I know for most people like me it’s unaffordable.”

(Woman, 24, blind, urban)

“It made me feel sad and angry that I lost independence to even do these simple things…That’s why I purposely designed it as you can see to suit my needs for bathing and toileting, these were the first things I learnt to do on my own again.”

(Man 45, partial paralysis, rural)

These individuals were well educated and relatively wealthy compared with other study participants. These factors are likely to have enabled them to adapt their environment to their needs.

All of the remaining disabled respondents reported at least one WASH access challenge. Seven individuals ranked at least one WASH related activity within the three biggest challenges they faced in daily living and one reported that all of their top three challenges were WASH related. Water access was the most commonly reported challenge among disabled respondents (Table 4). These challenges included difficulties accessing stored water for drinking, bathing and handwashing, as well as barriers to collecting water.

**Table 4:** Number of disabled respondents experiencing each of the daily challenges.

Disabled Respondents (n = 11)	
Type of challenge	Number of respondents*
Getting water or getting enough water	9
Mobility limitations (e.g. unable to travel independently or to go to certain places)	8
Leisure limitations (e.g. socialising less or being unable to participate in certain social activities)	6
Bathing	5
Challenges specific to impairment that affect all aspects of daily living (e.g. seizures, confusion, impairment deterioration)	5
Maintaining relationships (e.g. unable to build new relationships or feeling socially isolated)	5
Reliance on others in daily tasks	5
Getting to or using the toilet in an acceptable way, managing urination and defecation and/or disposal of faeces.	4
Perceived loss of opportunities (e.g. education or career opportunities)	4
Money concerns	3
Cleaning and household tasks	3
Farming	3
Cooking	3
Clothes washing and ensuring there are always enough clothes	2
Access to services (e.g. unable to travel to a health centre)	1
Soap availability	0
Eating	0
Information and communication	0
Total WASH-related responses	20 (30%)

*Note, in this table challenges are listed in descending order according to the number of respondents who reported each challenge as being among their important daily challenges. This does not correspond to the priority ranking of challenges given by individual respondents.

After ranking their free-listed challenges, all respondents were asked specifically for any other WASH related challenges they faced. Five individuals added issues relating to toileting and/or bathing to their list and in most cases chose to insert them fairly high up in their ranking order. Among WASH challenges, toileting was generally ranked as a high priority on individual lists.

When disabled respondents were asked why WASH-related challenges ranked high on their lists. They explained that it was because limited WASH access:

i) led to increased health risks,

“I am very sad when it comes to the way I use the toilet because I can catch infectious diseases in these places, it has implications on my health.”

(Man, 46, double amputee. Peri-urban.)

“The toilet problem is a serious problem because if I open bowels or suffer from diarrhoea, I defecate where I am and they clean me…it becomes a health problem to other people around me.”

(Woman, 78, arthritis and spinal curvature. Peri-urban.)

Ii) increased dependency

“It makes me feel sad and angry that I have lost independence to even do these simple things.”

(Man, 46, amputee. Peri-urbanl.)

and iii) had negative consequences for their self-image.

“Let’s say within the night I have seizures but there is limited water. That means I cannot wash my body and clothes properly so this is a major challenge. It means there are a number of people who you can tell have epilepsy because of how they smell….It’s a tough moment for me.”

(Man 27, Epilepsy. Urban.)

“The way I get water and use the toilet makes me feel different.”

(Woman, 20, partial paralysis. Rural.)

Table 4 shows the number of respondents reporting each type of challenge, while Table 5 shows the number of caregivers reporting each type of challenge. The daily living challenges identified by respondents were grouped into 18 categories by the lead author.

**Table 5:** Number of caregivers reporting each of the daily challenges.

Caregivers (n = 3)	
Type of challenge	Number of respondents*
Bathing	3
Eating	3
Clothes washing and ensuring there are always enough clothes	2
Soap availability	2
Getting to or using the toilet in an acceptable way, managing urination and defecation and/or disposal of faeces.	2
Challenges specific to impairment that affects all aspect of daily living (e.g. seizures, confusion, impairment deterioration)	2
Information and communication	2
Reliance on others in daily tasks	1
Getting water or getting enough water	1
Mobility limitations (e.g. unable to travel independently or to go to certain places)	0
Leisure limitations (e.g. socialising less or being unable to participate in certain social activities).	0
Maintaining relationships (e.g. unable to build new relationships or feeling socially isolated)	0
Perceived loss of opportunities (e.g. education or career opportunities)	0
Money concerns	0
Cleaning and household tasks	0
Farming	0
Cooking	0
Access to services (e.g. unable to travel to a health centre)	0
Total WASH-related responses	10 (56%)

*Note, in this table challenges are listed in descending order according to the number of respondents who reported each challenge as being among their important daily challenges. This does not correspond to the priority ranking of challenges given by individual respondents.

For caregivers of people with intellectual impairments the availability of sufficient soap and sufficient clothes emerged as important concerns. These challenges were not reported by disabled respondents. For two of the three caregivers WASH issues comprised all of their top three challenges of daily living. None reported that the person they cared for had problems accessing water, but access to sufficient quantities of water was seen as a problem for the family in relation to increased bathing needs. Carers explained that these issues ranked high among their priorities because they took up a substantial amount of their time, had a physical and psychological impact on household members and presented challenges for long term care.

“These things are very important because this child needs to be clean and well taken care of all the time, most of my attention goes into bathing him.”

(Carer of a boy, 8, with cerebral palsy. Rural.)

“It takes a lot of effort from all of us. For every 5 buckets of water, we would use three or four on bathing her and we have to carry her to the bathroom, it’s a strain now because she is heavy.”

(Carer of a girl, 14, with intellectual impairment. Rural.)

“It hurts me a lot…it’s kind of psychological because other children of that age can clean themselves and bath themselves but for him I think I will probably have to help him do that for the rest of his life.”

(Carer of a boy, 13, with intellectual impairment. Peri-urban.)

The three respondents who participated in the emotion mapping exercise each reported that they were unhappy in some of the WASH environments at their home. The first participant, a young man with an intellectual impairment, was unhappy in the toilet. He explained that he found it difficult to squat down in the toilet. His carer was surprised to hear about his unhappiness, explaining that “*I did not know about this issue until now*, *but I think now I will try and make a seat for him*.” Later in the interview the participant explained that sometimes he didn’t like bathing either but was often forced to by his carer. The second participant, a young boy with a physical impairment, explained that although he felt happy when he was pumping water he hardly ever went to the water point because it was difficult for him to get to. He also reported being unhappy in the toilet and the bathroom because they were smelly places and they made him feel embarrassed because he had to use them differently. The last respondent, a girl with an intellectual impairment, reported that the tap near her house was an unhappy place because it was hard for her to turn it on. The other environments (bedroom, living room, kitchen and outside space) were reported to be happy places.

### Categorisation of WASH access Barriers

Reported and observed barriers were categorised by the lead author using the physical, social and institutional categories [4, 12–16] and disaggregated by the nature of the respondent’s impairment (Table 6). No two individuals in this study faced the same set of barriers. The barrier classification presented is subjective and barriers often fitted more than one category. For example people with intellectual impairments were often excluded from community events on the assumption that they could not understand or contribute to them or would disturb the process. This barrier was classified as institutional because it is something that implementers of WASH programs may enable rather than actively challenge but is also a social barrier maintained by societal attitudes and values.

**Table 6:** Categorisation of reported and observed barriers.

	Environmental / Physical Barriers	Social Barriers	Institutional Barriers	Barriers Associated with Body Function
**Physical Impairment**	(1) Long distances to toilets, bathrooms and particularly water points. (2) Standard pit latrines may be difficult to use due to problems squatting. (3) Navigating slippery or uneven surfaces. (4) Water stored in large containers making it difficult to access (e.g. for people with restricted growth) or at low height making it difficult to bend and lift (e.g. for people with joint pain or arthritis). (5) Hard to reach taps, pumps and basins (e.g. for people with restricted growth or those who have to crawl). (6) Uneven ground (e.g. for people using wheelchairs, for those who have limb or joint pain and for people with lower limb amputations). (7) Infrastructure that includes steps, rims, narrow doors or a lack of space in the facility. (8) Stagnant water around water points, bathrooms and toilets. (9) Individuals may have to touch surfaces in toilets and bathrooms with their hands/other body parts in order to adapt to common toilet and bathroom designs (e.g. due to crawling or to maintain balance). (10) This is a particular issue if facilities are left unclean. (11) Handwashing may be problematic as clean hands have to be placed back on to crutches, wheelchairs or surfaces that are unclean.	(1) Family members may provide a high level of assistance rather than consulting the individual about their preferred WASH solution or exploring options that promote independence. (2) In some settings epilepsy and albinism are seen as contagious and could potentially be transmitted through WASH related practices (e.g. sharing water). (3) Individuals are often isolated and stigmatised and are therefore less likely to participate or speak up about their situation. (4) People with epilepsy or mental health challenges may not be permitted to collect water as others do as there is a perceived risk to their safety. (5) People with restricted growth are sometimes not consulted in the same way as other adults (treated as children) so their WASH needs may be overlooked. (6) The community may be unaware of the existence of disabled individuals in their community as they may be confined to the home.	(1) Some individuals less connected to services and disability networks (e.g. people with joint pain, mental health issues and chronic illness). (2) Events delivered at the community level prevent the attendance of people who cannot travel outside the home. (3) Community events are often not conducive to enabling disabled people to attend (e.g. long duration, type of seating, accessibility of venue, etc.). (4) People with epilepsy are often not identified as disabled by implementers. (5) Disabled people may require persuasion to participate in community events (e.g. people with mental health issues). (6) People with albinism may not attend community events due to concerns about sun exposure.	(1) People with physical impairments, including restricted growth and joint pain may have difficulty carrying water or can only carry smaller volumes of water. (2) People with albinism may be unable to collect water during daylight hours due to the sunlight exposure and may not always have enough during the day to meet their needs. (3) Older people or those with paralysis may experience incontinence or lack of sensation. (4) Women may have difficulty managing their periods or experience discomfort during this time due to often being seated (e.g. wheelchair users). (5) People with limited mobility cannot independently go to the toilet or bathe.
**Visual Impairment**	(1) Pathways to water points and toilets are unmarked/ bumpy/have obstacles. (2) Toilets may be unclean resulting in dirtying of clothes, hands, etc. (3) Difficulty finding the hole when using pit toilets—large holes also a risk. (4) Difficulty locating soap.		(1) WASH programs often rely on visual elements and materials.	(1) Women may be unable to respond to the visual cues of menstruation, making periods harder to manage and a source of embarrassment. (2) Difficulty carrying water/only able to carry smaller volumes of water which means they are not always able to meet their needs.
**Hearing impairment**		(1) May be thought of as unintelligent or not having an opinion because it is more challenging to communicate. (2) Privacy may be harder to maintain as individual’s can’t hear others approaching to use the toilet/bathroom.	(1) Often not invited nor able to participate in WASH related community events. (2) Less connected to services and disability networks. (3) WASH programs are often verbally presented	(1) May be unable to communicate their WASH needs.
**Intellectual/ cognitive impairment**	(1) Squatting over a standard pit latrine can be difficult. (2) Some types of taps and pumps are difficult to use. (3)Uneven surfaces are difficult to cross. (4) Distance to toilets or bathrooms make it harder for the individual to locate (e.g. for people with dementia).	(1) Mental health issues are often not treated as disabilities and the WASH needs of these individual’s may be overlooked. (2) Families often do everything for the individual rather than trying to develop processes that will enable the individual to maintain independence (e.g. for people with dementia or intellectual impairment). (3) Intellectual impairment might be understood as a curse.	(1) Presumed to not be able to contribute usefully to community events. (2) Less connected to services and disability networks. (3) Excluded from community events on the assumption that they cannot understand or contribute and/or will disturb the process.	(1) People with dementia may have trouble remembering routes to the toilet, bathroom or water point. (2) People with dementia may forget when they last used the toilet or bathed. (3) May not be able communicate their WASH needs. (4) May experience incontinence and not be able to independently go to the toilet or bathe.

Some of the barriers identified did not fit into the categories which were based on a social model of disability. Many such barriers occurred as a direct result of the individual’s impairment and are described in Table 6 as ‘barriers associated with body function’. All of the institutional barriers mentioned by participants related to community-level participation. No participants referred to barriers relating to higher-level policies, systems or institutions.

### Urban / rural differences

The way in which water and sanitation are provided can vary between urban and rural contexts with implications for the barriers faced by disabled people. For rural respondents piped water was not available. One rural respondent used an unimproved water source near their home but most relied on communal handpumps. These had minimal usage costs but were rarely close to the house. Thus physical barriers associated with distance and the need to carry water emerged as important concerns for disabled people in rural areas. In urban areas piped water was available for those with the ability to pay. Respondents with piped water reported no barriers to water access. Most urban respondents without piped water were reliant on water purchased from kiosks which were perceived by disabled people to be too far from their houses (based on observations and anecdotal evidence these were generally within 500m). As a result they faced barriers associated with distance to the water point as well as physical barriers associated with the design of the kiosks (poor drainage, types of taps, steps and rims). Several respondents reported this led to a double financial burden which made them compromise on quantity of use.

“In my community there are kiosks where they sell water, so like the way I am, using a wheelchair, I can’t take a bucket to buy water … I pay twice, once for the water itself and once for the one carrying water for me.”

(Woman 35, paralysis. Urban)

Those who could not afford to use kiosks relied on unimproved water sources such as rivers and shallow, hand-dug wells. These participants were more concerned by the consequences for their safety and health. One example of this was a household with a boy of 14 who had an intellectual impairment. The boy was incontinent and required family members to wash him and his clothes regularly. The amount of water and soap they required as a household was much greater than neighbouring families. Consequently the family was unable to afford their piped water bills and had to make water and hygiene compromises.

“Normally we all have diarrhoea at least twice a week…It’s because we drink the water straight from the river. We used to have piped water to a tap near the house and when we were using that water we didn’t have diarrhoea as much…it’s hard because in town if you don’t pay for piped water then you have to get water from the river, there are no water pumps where you can get water for free like in other places. It’s really not healthy to drink out of the river. Just today there was a dead dog upstream.”

(Caregiver of a boy, 14 with an intellectual impairment. Peri-urban)

A comparison of observations in rural and urban settings revealed that sanitation access and use was also likely to be more challenging for urban dwellers. The quality of latrine superstructures were observed to be poorer in urban areas, providing little support for individuals with mobility impairments. Latrines in urban areas were also more commonly shared between several families, making them less clean and less well suited to the needs of a disabled person. Drainage was also observed to be a common problem in urban areas.

### Gender

Traditionally, Malawian men and women play different social roles. This affects performance and perceptions of WASH activities. One respondent highlighted an association between marital status and water access.

“In our culture for woman to be a woman, you need to be able to fetch water. If a man is looking for a wife he wants a woman who is able to draw water and collect firewood, that’s what being a good wife is.”

(Woman, 47, paralysis. Urban)

WASH demonstrations and observations of two respondents with physical impairments in a poor area of Lilongwe provided another example of gender differences relating to water access. The male respondent was married and gave the following summary of his water access.

“Because I am happily married it’s my wife who does the water collection. It’s normally easy for me to access the water as she brings it close to me. I am not happy with the life I live because I would like to be more independent. It worries me that sometimes I might need to access water but my wife might not be there… but this never really happens because my wife is so close and so supportive to me.”

(Man, 40, paralysis. Urban)

The other respondent was a 24 year old female. She explained that “even though I live with my brother, he does not help draw water so I have to do it all myself”. It was observed during her WASH demonstration that as an amputee it was difficult for her to cross the uneven, muddy ground and lean down to draw water from a well before having to haul the bucket onto her head. None of the disabled men in the study reported collecting water on a regular basis yet 10 of the disabled women were responsible for getting water.

Menstruation also imposes different WASH needs on women which can be more pronounced for disabled people. One respondent, a local government employee, explained that she frequently met girls with disabilities who have been encouraged to go to school but who then drop out when they begin menstruation:

“When they are in periods they don’t go to school, they stay one week, two weeks at home… Because imagine, if you are like the way I am [a wheelchair user] sitting down the whole time you are in periods, I wouldn’t be able to hear anything the teacher is teaching because I would be worried that maybe things are wet and I need to change.”

(Woman 47, paralysis. Urban.)

Menstruation challenges were found to be a source of shame, discomfort and worry for all of the disabled women interviewed. Menstruation was particularly challenging for women with visual impairments, for whom it was hard to identify when their period began and ended, for wheelchair users who experience discomfort due to being always seated and for people with intellectual impairments who were often not able to manage their periods independently.

“The challenge during menstruation is that all I can do is sit here. I do feel hot during that time and often the blood can go out through the clothes and it is embarrassing for me.”

(Woman, 35, paralysis. Rural.)

“It’s a challenge because of my visual impairment because I am caught unaware that I have messed up my beddings. It’s an embarrassment because I assume that this may be seen by others. Sometimes you can tell with the signs that you are going to start menstruating but it’s more difficult when you don’t get those signs and that concerns me hygienically.”

(Woman, 43, blind. Urban.)

### Wealth and education

Although poverty was not measured empirically in this study, disabled people who appeared less poor (based on observed assets and housing) were found to have better WASH access. Higher levels of education and regular employment meant that individuals were more able to connect with services, participate in community events, be aware of policy and community structures and be able to independently mitigate their WASH challenges.

“Now I am one of the lucky ones, in my house I have been able to pay for piped water to be installed and I built a toilet inside so I have no problems now, but I am a special case, the majority of people with disability in this country don’t have this opportunity, they can’t afford it.”

(Woman, 27, blind. Urban.)

“I imagine somebody who is not working might experience more stigma and more challenges than me. I am accepted because am educated, I am accepted because I make money for myself. But I understand that those who are in the villages are experiencing more stigma up to this day.”

(Man, 37, albinism. Peri-urban.)

Poorer disabled people were more likely to rent accommodation and feel disempowered or discouraged from improving their household environment.

“The best thing for me would be to have a permanent place then I could build my own toilet and bathroom. Here [in rented accommodation] I could be chased away any time so any changes I make would be a waste of money. So it is about money, but in this situation it’s also more complex than just having the money.”

(Man, 46, double amputee. Peri-urban.)

## Discussion

Access to improved water supply and sanitation as well as safe hygiene practices remains low across low-income countries. Given the challenges in addressing these issues for many low-income households it is not surprising the situation faced by disabled people, disadvantaged both physically and socially, is even worse. Previous studies have pointed to the importance of water [22] and sanitation [4, 23] in the lives of disabled people. The current study explored how these issues ranked in relation to other priorities of daily living. The study found that WASH access was a prominent concern for disabled people and one that negatively affected their lives and those of their carers. WASH-related tasks were perceived as a priority because of their association with self-esteem, independence, and also because of the health and the productivity consequences they have for individuals, their households and the broader community.

Literature on WASH and disability has frequently focused on WASH barriers. However, respondents in the current study highlighted not only barriers to WASH access but also the WASH *needs* associated with disability and the *consequences* experienced as a result. Examples of these are given in Table 7.

**Table 7:** Definitions of WASH barriers, Needs and Consequences.

	Definition	Examples
**Barriers**	Anything that: a) reduces access to and/or use of WASH facilities; b) causes use of WASH facilities in a way that is unacceptable; c) limits or prevents participation in WASH programs; d) reduces access to information about WASH.	***Physical/environmental barrier*:** Lack of smooth, flat surfaces make getting to toilets, bathrooms and water points difficult. ***Social barrier*:** Intellectual disability is understood as a curse and community members are unlikely to try and support such individuals. ***Institutional barrier*:** Not invited or able to participate in WASH related community events
**Needs**	Anything that requires an individual to use WASH facilities differently, or more or less frequently due to their personal characteristics and impairment.	***Greater WASH needs*:** A household where there is a person who is incontinent will need to collect more water than other households. ***Additional WASH needs*:** Some people with paralysis use catheters. ***Different WASH needs*:** People with albinism may need to collect water at different times than others, meaning they may not always have enough water during the day to meet their needs.
**Consequences**	The experiences associated with WASH barriers and needs including increased pain and impact on health and self-esteem.	***Physiological consequences*:** Joint pain resulting from carrying water. ***Health-related consequences*:** Diarrhoea resulting from difficulties in maintaining hygiene. ***Social consequences*:** WASH access barriers result in personal hygiene compromises which result in further stigmatism. ***Institutional consequences*:** Reduced job or education opportunities due to inability to access WASH facilities in a way that is acceptable and dignified.

Removing or reducing barriers is likely to be the primary means by which WASH interventions might achieve greater inclusion for disabled people. However, as shown previously [24] the current study found that people with impairments may have different or greater WASH needs than others in their community. Implementers may therefore need to consider how to achieve equitable rather than equal WASH access. This may require thinking beyond adaptive technologies to include community support (e.g. assistance to collect greater amounts of water) and hygiene promotion tailored to specific needs (e.g. providing guidance to carers of people with severe impairments on how they can mitigate their unique hygiene challenges).

Understanding WASH consequences provides important context to this issue. It can inform broader agenda setting by highlighting the measurable impact that inaccessible or unacceptable WASH has on the lives of disabled people and the wider community including its association with productivity, disease transmission and self-esteem.

This study highlighted the variation that existed between people with disabilities with regard to the WASH barriers they faced. Some of this variation was related to the nature of individual impairments. Respondents with hearing impairments, epilepsy and mental health challenges were not found to face physical barriers while those with physical disabilities faced predominantly physical barriers. Institutional barriers were faced by all to some extent. However, people with sensory impairments, such as visual and hearing impairments, were found to be most disadvantaged when it came to participation in WASH events and access to information. Social barriers were found to affect people with most types of impairments. However, people with intellectual impairments, epilepsy and albinism were more likely to be affected by social barriers associated with cultural beliefs. People with only physical impairments were less likely to face social barriers.

The distinction between social, physical and institutional barriers is useful because it points towards different courses of action to facilitate WASH access. However, the analysis of barriers also revealed challenges arising as a direct consequence of an individual’s impairment. For example, people with hearing impairment may have difficulty in communicating WASH needs. Some people with physical impairments struggle to carry enough water to meet their needs or experience pain when they do so. People with intellectual impairments or severe physical impairments may experience incontinence. Consequently households in which such individuals reside are likely to need more water to mitigate the associated hygiene challenges. For some individuals with incontinence, toilet use may not be a feasible expectation.

Unlike the social model of disability, the ICF framework explicitly acknowledges that an individual’s impairment can directly contribute to reduced participation in WASH activities. Furthermore this reduced participation may not be addressed entirely through changes in infrastructure, social attitudes or institutions. Recognising this may be an important step towards identifying and creating appropriate support for individuals and their carers.

The extent to which impairments translate into disabilities depends not only on characteristics of the impairment but also characteristics of the individual and their environment [25]. This study, albeit with a small sample, explored the variation in barriers associated with gender, education, wealth and location (urban or rural). Being female and having limited resources and education were factors that made WASH barriers more pronounced. These findings are consistent with patterns of inequitable WASH access in the non-disabled population [26]. Our study supports earlier findings with respect to disabled women and urban and rural differences [4, 27].

Given the diversity in individual contexts, needs, barriers and impairments this research could not provide a comprehensive picture of the ways in which these factors interact to disable people with respect to WASH access. Further work with larger and more homogenous samples is needed to understand specific risks of disability associated with particular combinations of impairment and external factors. Individuals with mental health challenges are one example of a group that have been relatively neglected in the WASH literature. Further work might also seek to quantitatively measure the specific health and economic consequences faced by disabled people as a result of poorer WASH access.

The diversity of WASH barriers, needs and consequences is a challenge for implementers as well as researchers. Solutions need to address a variety of needs and this may contrast with the use of standardised approaches to deliver WASH interventions at scale. There are low-cost, simple hardware adaptations that can address some physical barriers for individuals with certain impairments [28]. However, there are other impairments for which, when combined with poverty and poor infrastructure, effective solutions are more challenging. One important approach in the face of this diversity continues to be efforts to make WASH interventions more inclusive with respect to listening to the needs of disabled individuals [29]. The right to be included is perhaps the lowest common denominator cutting across individual variation. Interventions such as Community Led Total Sanitation, which are implemented at scale and follow a relatively uniform protocol, may offer opportunities for the addition of standardised steps to improve inclusiveness. Such an approach might allow for the generation of solutions that are context specific and responsive to the needs of the individual but still feasible to deliver at scale.

Upstream influences such as policies and institutional structures no doubt have an important role to play in creating or removing barriers to inclusion and to WASH access. Discussion of these barriers was not a focus of this study simply because they did not feature in the perceptions of barriers presented by respondents. This in itself may be indicative of a certain degree of exclusion of disabled people from the agenda setting process. Partnerships with key agencies beyond the WASH sector are also likely to play an important role in meeting the needs of disabled people. However the study did not explore this topic.

The research methods employed in this study helped generate data that would not have been achieved through in-depth interviews alone. Emotion mapping helped ensure the perspectives of children and people with intellectual impairments were not overlooked in favour of interviewing only their caregivers. The Photovoice method was described by participants as “*memorable*”, “*fun*” and “*unlike anything I have ever tried before*” and has subsequently provided advocacy opportunities for the perspectives of participants to be shared with a wider, non-academic audience [30]. WASH demonstrations and observation were found to be acceptable and helped facilitate discussions around taboo issues. The study also benefited from the input of disabled people, recruited as field assistants, to facilitate interviews and data collection during fieldwork. These people were able to use their own experiences to inform the design of the research tools and the scope of questioning. Their presence also helped to build rapid rapport with respondents. The research was also guided by the feedback generated through a stakeholder workshop at the inception of the project, which included disabled people and representatives of disabled people’s organisations as well as service providers.

## Conclusions

Documenting the range of WASH challenges faced by disabled people and identifying commonalities within this range may facilitate the development of innovative hardware and software solutions. This study took an initial step along this path in Malawi, exploring the utility of existing models of disability and experimenting with novel data collection methods in the process. Analysing the perspectives and experiences of disabled people through two dominant theoretical models of disability enabled this study to highlight WASH needs and challenges which are not fully addressed by WASH programming. Addressing these shortcomings and reaching out to the individuals who are currently excluded will be critical to achieving WASH access for all.
